# Eye-movement markers of mind wandering during reading: A meta-analysis

**DOI:** 10.3758/s13421-025-01797-8

**Published:** 2025-10-17

**Authors:** Diane C. Mézière, Niilo E. Hautala, Timo T. Heikkilä, Johanna K. Kaakinen

**Affiliations:** 1 Department of Psychology and Speech-Language Pathology, University of Turku, Assistentinkatu 7, Publicum 2nd Floor, Turku, Finland; 2 INVEST Research Flagship, University of Turku, Turku, Finland

**Keywords:** Eye movements, Reading, Mind-wandering, Meta-analysis

## Abstract

Mind wandering during reading has been extensively investigated, with multiple studies reporting differences in eye-movement behavior between mind-wandering and on-task reading. More recently, researchers have tried to use eye-movement measures to predict mind wandering *during* reading; however such indicators of mind wandering during reading have not yet been clearly identified. This article presents results from two studies. Firstly, we carried out a meta-analysis to identify eye-movement indicators of mind wandering during reading. From the initial search, abstracts from 140 articles were reviewed for eligibility, and 39 articles were included for full text reading and data extraction. Finally, we identified 16 individual datasets from 19 articles for which we could compute effect sizes. We calculated effect sizes for nine eye-movement measures: mean fixation duration, fixations count, first-fixation duration, gaze duration, total reading time, saccade length, skipping, blink count, and inter-word regressions. The results indicated that readers skipped more words and made fewer fixations during mind-wandering compared to on-task reading. Following these results, we carried out exploratory analyses with a dataset from our own lab to examine other possible explanations for this eye-movement pattern. Specifically, we investigated readers’ sensitivity to word frequency and length and readers’ use of corrective regressions. Results indicated that readers show reduced effects of word length and frequency on skipping behavior as well as a reduced use of corrective regressions during mind-wandering compared to on-task reading. Results and implications from both studies are discussed in the context of mind-wandering and reading research.

**Supplementary Information:**

The online version contains supplementary material available at 10.3758/s13421-025-01797-8.

## Introduction

Mind wandering has been used to refer to a variety of thought patterns that typically include instances in which thoughts are unrelated to the stimuli and/or task at hand (see Seli et al., 2018). In reading research, mind wandering is most often conceptualized as an unintentional lapse of attention occurring during a reading task (e.g., “mindless reading”; Smallwood & Schooler, 2013), and it is typically measured as instances of attention being either on or off the task. In such instances of mind wandering, attention is decoupled from the stimuli and/or task at hand (e.g., the text and/or the reading task), and tends to focus inwards, typically involving internal thoughts that may be related to past events (e.g., what happened yesterday) or the future (e.g., what you will do once you graduate), or involve thoughts related to other people (e.g., Ruby et al., 2013). Episodes of inattention to the stimuli and/or task at hand are often associated with negative impacts on task performance (e.g., Smallwood et al., 2004; but see Smallwood & Andrews-Hanna, 2013, for discussion on positive effects of mind wandering), particularly for tasks that put higher demands on sustained attention and/or cognitive load such as reading (e.g., see Randall et al., 2014, for a meta-analysis). Research suggests that mind wandering may also be intentional (Seli et al., 2016), in which case the lapse of attention is voluntary, and the contents of the thoughts during mind-wandering episodes may vary (e.g., Ruby et al., 2013). However, in the present meta-analysis, we will focus on the effects of unintentional mind wandering characterized simply by being off-task, as most research in mind wandering during reading has used this definition.

### Mind wandering during reading

One area of research in which the impact of mind wandering has gained a lot of interest is the study of reading. Indeed, while many theories of reading assume that the reader focuses their attention on the text content (see McNamara & Magliano, 2009, for a review), research suggests that mind wandering occurs around 20–40% of the time during reading (Schooler et al., 2004). Text comprehension is a complex cognitive task which requires sustained attention, from visual processing of the stimuli (i.e., identifying individual words) to higher-level processes that support language comprehension (e.g., building a representation of the text). Momentary inattention to the task can thus be expected to influence the different cognitive processes that support text comprehension.

The cascade model of inattention aims to explain how mind wandering during reading may impact text comprehension (Smallwood, 2011). According to the model, when attention is decoupled from the text, readers should be less sensitive to linguistic characteristics of the text such as word frequency, which in turn will impact a reader’s sentence and proposition processing and ultimately negatively impacts their representation and comprehension of the text. In line with these predictions, research on the influence of mind wandering on the cognitive processes that support reading suggests that mind wandering negatively affects reading at multiple levels of processing including early processes of word identification (Foulsham et al., 2013; Reichle et al., 2010; Smallwood et al., 2007; Smilek et al., 2010), sentence processing (e.g., Zhang et al., 2020), as well as higher-level discourse processes (e.g., inference making: Smallwood et al., 2008), which in turn negatively affects text comprehension (D’Mello & Mills, 2021; Franklin et al., 2011; Mooneyham & Schooler, 2013; Schooler et al., 2004; Smallwood et al., 2008; see Bonifacci et al., 2023, for a meta-analysis). In other words, when readers mind wander their attention is no longer on the text, hence leading to shallower processing of the text (e.g., shallow lexical processing) which ultimately leads to a less detailed and less accurate mental representation of the meaning of the text (i.e., situation model).

Recent work has aimed to identify markers of mind-wandering episodes in order to detect mind wandering as it occurs and develop interventions to refocus the reader’s attention to the text (e.g., Bixler & D’Mello, 2014, 2015, 2016, 2021). A great majority of this work has been carried out using eye tracking, which provides an online measure of cognitive processing during reading (see Rayner, 1998, for a review).

Research on mind wandering during reading suggests that eye-movement behavior does differ between on-task reading (in which the attention is focused on the text) and mind wandering (in which the attention is focused elsewhere), suggesting differences in cognitive processes involved in reading during mind-wandering compared to on-task reading (e.g., Reichle et al., 2010; Smilek et al., 2010). Much of the literature on the impact of mind wandering on eye-movement behavior during reading comes from cognitive psychology experiments in which readers are asked to report (typically following a prompt) whether they were mind wandering or on task. This field of research primarily examines two types of measures related to eye movements: fixations (i.e., instances where the eye remains mostly stationary), and saccades (i.e., fast movements through which the eye moves from one viewing location to another), with saccades including both forward movements in the text and backward movements to previous parts of the text (i.e., regressions). In addition, eye-movement research into mind wandering has considered both word-level or local measures which are calculated at the level of individual words (e.g., first-fixation duration: the duration of the first fixation made on a given word), and global measures which are calculated and often aggregated over the whole text (e.g., average fixation duration). Results from such studies suggest that mind-wandering episodes tend to be associated with longer text reading times (Foulsham et al., 2013; Nguyen et al., 2014), longer average fixation durations (Foulsham et al., 2013; Gwizdka, 2019; Oyarzo et al., 2022), as well as higher rates of word skipping (i.e., not fixating a word; Nguyen et al., 2014; Zhang et al., 2020) and blinking (Frank et al., 2015; Oyarzo et al., 2022; Smilek et al., 2010) compared to on-task reading. These findings suggest that while readers may fixate fewer words while they are mind wandering, they make longer fixations on average, thus leading to longer overall reading time, although some studies find no such relationship between fixation durations and mind wandering.

However, the finding that readers make longer fixations during mind-wandering compared to on-task reading is less apparent in studies that report word-based or local fixation measures (i.e., measures calculated for individual words) compared to studies reporting global measures (i.e., measures calculated at the whole-text level). Indeed, results for word-level fixation measures such as gaze duration (i.e., the sum of all fixations made on a word during first-pass) or total reading time (i.e., the sum of all fixations made on a word including re-readings) are widely inconsistent across studies, as some findings suggest that mind wandering is associated with longer gaze durations and total reading time (e.g., Reichle et al., 2010), while others find the opposite (Nguyen et al., 2014), or report no difference between mind-wandering and on-task reading (e.g., Frank et al., 2015; Schad et al., 2012; Zhang et al., 2020). Similarly, findings on the effect of mind wandering on saccadic movements during reading (i.e., movements forward or backward in the text) are also contradictory, with some studies finding that readers make longer forward saccades during reading, which would be in line with the finding that readers also skip more words (Gwizdka, 2019; Nguyen et al., 2014), while others find the opposite (Oyarzo et al., 2022), or do not find any impact of mind wandering on saccade length (Foulsham et al., 2013). Hence, while findings typically indicate that eye-movement behavior does differ between on-task reading and mind wandering, the contradictory findings across studies renders the identification of possible useful predictors of mind wandering difficult.

More recently, however, this predictive relationship between eye-movement measures and mind wandering has been investigated more directly in studies using machine-learning methods to predict mind-wandering episodes from eye-movement behavior during reading. Such studies show that features of eye-movement measures can be used to predict mind wandering with above-chance accuracy (59–72%: Bixler & D’Mello 2014; 74%: Bixler & D’Mello, 2015; 60–72%: Bixler & D’Mello 2016), and are generally in line with other work suggesting that mind wandering is typically associated with fewer and longer fixations (Bixler et al., 2015; Faber et al., 2018). In addition, results showing the most useful predictors of mind wandering suggest that global measures tend to be more useful in predicting mind wandering compared to word-based measures, as word-based measures typically only minimally improve models over and above models that only include global measures as predictors. In addition, these studies suggest that measures of the spread and shape of the distribution of eye-movement measures (e.g., standard deviation, kurtosis) are useful predictors of mind wandering as well as the more commonly used measures of central tendency reported in other works (e.g., mean fixation duration). While the importance of measures of variance in eye-movement behavior suggests that mind-wandering episodes may be reflected in the regularity of gaze behavior, much of these measures are much harder to interpret in relation to cognitive processing (e.g., fixation duration kurtosis). Thus, while such studies provide evidence that eye-movement measures can be used to predict mind wandering during reading, and provide additional information as to which measures are most useful in accurately predicting mind wandering, they tend to be less informative about the strength and direction of the relationship between individual eye-movement measures and mind wandering as such methods do not necessarily tell us *how* the model uses the eye-movement features to predict mind wandering.

Taken together, results from both cognitive psychology experiments and machine learning studies show that eye-movement behavior is influenced by mind wandering during reading compared to on-task reading, and that eye-movement features can be used to predict mind wandering during reading. Nevertheless, to date, eye-movement markers of mind wandering during reading have not been clearly identified, thus rendering the identification of mind-wandering episodes *during* reading difficult. In this article, we present results from two studies. First, we present the results of a meta-analysis on the differences in eye-movement behavior between on-task reading and mind wandering, with the aim of identifying possible eye-movement markers of mind wandering during reading. Second, we present results from exploratory analyses that aim to shed light on the results of the meta-analysis.

### Aim and research questions

The principal aim of this article is to identify potential eye-movement markers of mind wandering during reading. In a meta-analysis, we first identified eye-movement measures commonly reported in experiments investigating eye-movement behavior during mind wandering compared to on-task reading. We then calculated effect sizes for these measures comparing mind-wandering episodes to on-task reading in order to identify candidates for useful predictors of mind wandering during reading. Based on these results, we conducted exploratory analyses on a dataset from our own lab to further inform the relationship between eye movements and mind wandering by exploring possible explanations for the results of the meta-analysis. This article aims to answer two main research questions:

1. How is mind wandering reflected in eye-movement measures during reading?
2. What differences in cognitive processing and reading behavior are reflected by eye-movement behavior indicative of mind wandering during reading?

## Study 1: Meta-analysis

Research on the impact of mind wandering, when defined as “task-unrelated thought”, shows that mind wandering is typically associated with poorer reading comprehension outcomes. As a result, researchers have investigated the possibility of detecting such mind-wandering episodes using eye-movement measures (e.g., Bixler & D’Mello, 2014, 2015, 2016; Bixler et al., 2015; Faber et al., 2018; Gwizdka, 2019), which could provide an online measure of lapses of attention during reading. However, as noted earlier, such eye-movement markers of “mindless reading” have not yet been clearly identified. Hence, the principal aim of this meta-analysis was to identify possible candidates for eye-movement markers of mind wandering during reading.

### Method

#### Inclusion and exclusion criteria

**Fig. 1:**
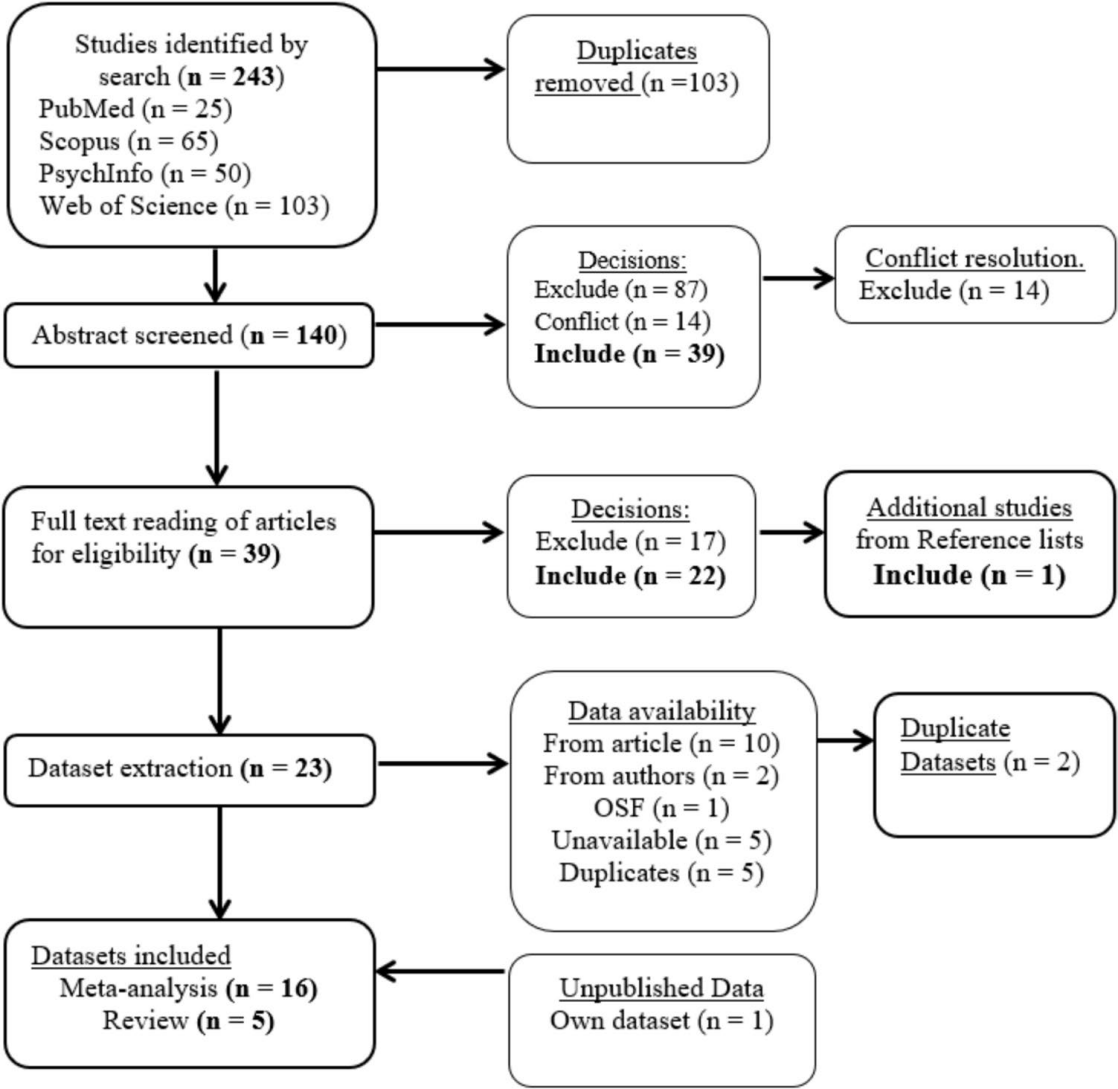
Article selection process

A systematic search was carried out following the Preferred Reporting Items for Systematic Reviews and Meta-Analyses (PRISMA) guidelines (Moher et al., 2010). An initial search was conducted using the following databases: PsychInfo (EBSCO), PubMed, Web of Science, and Scopus. This search was conducted in September 2023. Search terms included common-used terms for mind wandering when defined as task-unrelated thoughts (“mind-wandering,” “day-dreaming,” “self-generated thoughts,” “mindless reading,” “zoning out,” “tuning out,” “transportation,” “stimulus independent thought,” “task-unrelated thoughts”) as well as restricted the search to the use of eye-tracking methodology (“eye-tracking,” “eye movements,” “gaze behavior”) and to reading as the main task (“reading,” “text comprehension”). All search terms were included at once (e.g., “mind-wandering” OR “day-dreaming”) using the term AND between groups of terms to restrict the search to articles using eye-tracking technology to investigate mind wandering during reading. In addition to this initial search, we looked for additional studies in the reference list of included articles, and contacted authors about possible unpublished datasets that we should include. None of the contacted authors reported any unpublished dataset that should be included in our meta-analysis. However, we did include one unpublished dataset from our own laboratory. The article search and selection process and outcome is illustrated in Fig. 1.

The initial search for publications yielded 243 papers across databases. After removing duplicates, 140 article abstracts were reviewed for eligibility independently by two coders (first and third authors). Articles were excluded if they did not report any experimental data (e.g., review), did not include reading as at least one of the tasks, did not investigate mind wandering (e.g., mindless reading studies using the masked reading paradigm), or did not report eye-movement data (e.g., only reading times reported). Coders initially had conflicting opinions on the eligibility of 14 papers which were resolved by reading the articles in full by both coders. These 14 articles were ultimately excluded by both reviewers with 100% agreement. Following this initial exclusion round, 39 articles were read in full to further assess eligibility. Exclusion was based on the same criteria as for the abstracts, such that articles were excluded if they did not report experimental data, did not include reading as a task, did not investigate mind wandering or did not report eye-movement data. Of those, 22 articles were included for analysis, and an additional article was included from the reference lists of the articles read, for a total of 23 articles. We then extracted data for all eye-movement measures reported in these articles. In cases where a dataset was reported in more than one article, duplicates of the datasets were not included. For articles in which more than one experiment was reported, the data from each eligible experiment was treated as a separate dataset. In cases where the information reported in the article was not sufficient to calculate effect sizes, the authors were contacted to ask for either the dataset or descriptive statistics (mean and standard deviation) for the eye-tracking measures reported. Lastly, we included one (not yet published) dataset collected in our own lab that matched our inclusion criteria. In total, data could be extracted from 16 datasets across the 23 articles.

### Data collection

#### Eye-movement measures

We identified possible outcome variables (i.e., eye-movement measures) of interest according to two criteria. First, we identified measures that were commonly reported across datasets. As the measures reported across studies varied, we included only measures for which data were available from at least three datasets (see Valentine et al., 2010, for a discussion on the minimum number of studies necessary for a meta-analysis). Secondly, we focused on eye-movement measures that are commonly reported in the eye-tracking and reading literature to ensure a plausible interpretation of any effect found. We thus identified and computed effect sizes for 11 measures that matched our inclusion criteria, namely six global measures (i.e., measures calculated at the whole-text or analysis-window level in instances where the data were analyzed over a specific time window prior to answering the prompt) and five word-level measures (i.e., measures calculated for each word). Our six global measures were:

1. Mean fixation duration (the average fixation duration across all words in the text/window);
2. Mean saccade length (i.e., the average length of saccades made across the whole text/window);
3. Skipping^1^ rate (i.e., the proportion of words that were never fixated during reading);
4. Blink count (i.e., the number of blinks during reading the whole text/window);
5. Trial-level fixation count (i.e., the number of fixations made during the whole trial/window); and
6. Trial-level total reading time (i.e., the total time a participant took to read the passage/sentence).

In addition, we calculated effect sizes for the following word-level measures:

(7) First-fixation duration (i.e., the duration of the first fixation made on a word);
(8) Gaze duration (i.e., the sum of all fixations made on word before it is exited to the right or the left);
(9) Total reading time (i.e., the sum of all fixations made on a word including re-fixations);
(10) Fixation count (i.e., number of times a word was fixated); and
(11) Inter-word regressions count (i.e., number of look-backs made from a word to a previous word in the text). These measures and their definitions for each article are provided in Table 1.

As indicated in Table 1, the name of the measure used by some authors did not always match across studies or with the most commonly used terms in the literature. Hence each variable definition was carefully checked and validated by one of the authors to ensure that the measures were the same across datasets.

**Table 1:** Eye-Movement Measures Definitions

Measure	Article	Variable Name	Variable Definition
Mean Fixation Duration (*n* = 6)	Faber et al. (2020)	mean fixation duration	“*averaging across fixations […] in each time window*” (p. 1208)
	Foulsham et al. (2013)	fixation duration	“*average duration of all fixations on the sentence*” (p. 55)
	Gwizdka (2019)	average fixation duration	mean of fixation duration.
	Mézière et al. (2025)	mean fixation duration	mean of all fixations
	Oyarzo et al. (2022)	fixation duration	average fixation duration
	Smilek et al. (2010)	average fixation duration	average fixation duration
Mean Saccade Length (*n* = 6)	Faber et al. (2020)	saccade amplitude	“*averaging across […] saccades in each time window*” (p. 1208)
	Foulsham et al. (2013)	saccade length (letters)	“*average saccade length (excluding regressions)”* (p. 55)
	Mézière et al. (2025)	saccade amplitude	mean saccade amplitude
	Nguyen et al. (2014) Exp 1 &2	saccade length in characters	“*variables were averaged across all words within a passage*” (p.278)
	Oyarzo et al. (2022)	average saccade length	“*length of saccade trajectories*” (p. 10)
Skipping (*n* = 7)	Bixler and D'Mello, 2016		
	Mézière et al. (2025)	skipping	proportions of words never fixated during reading
	Nguyen et al. (2014) Exp 1 & 2	skipping	“*variables were averaged across all words within a passage*” (p.278)
	Schad et al. (2012)	word skipping	Not specified, proportion.
	Zhang et al., (2020) Exp 1 & 2	skipping	“*binary variable indicating whether the analysis region was not fixated on throughout the trial*” (p. 4)
Reading Time (*n* = 3)	Foulsham et al. (2013)	reading time	“*the duration that the sentence was displayed […] terminated by* *a* *saccade beyond the rightmost character*” (p. 55)
	Mézière et al. (2025)	total time trial	Total time taken to read the paragraph
	Steindorf and Rummel (2020)	reading time	*“time it took a participant to read a* *given target sentence”* (p. 169)
Blink Count (*n* = 7)	Danckert et al. (2018)	blink rate	average blink rate
	Faber et al. (2018)		
	Faber et al. (2020)	WindowBlinks	number of blinks in window
	Mézière et al. (2025)	blink count	total number of blinks during trial
	Oyarzo et al. (2022)	number of blinks	“*total amount of blinks*” (p.6)
	Smilek et al. (2010)	blink rate	“*blinks/5 s*” (Fig. 1. b, p.787)
	Steindorf and Rummel (2020)	blink count	“*number of observed occurrences of [blinks] during a given target sentence*” (p.169)
Fixation Count Trial (*n* = 6)	Faber et al. (2020)	Number of fixations	“number of fixations […] in each time window” (p. 1208)
	Foulsham et al. (2013)	fixation count	sentence-level
	Gwizdka (2019)	fixation count	whole time window
	Mézière et al. (2025)	fixation count	total number of fixations in trial
	Smilek et al. (2010)	fixation frequency	count over whole time-window
	Steindorf and Rummel (2020)	fixation count	*“number of observed occurrences […] during a given target sentence”* (p.169)
First-Fixation Duration (*n* = 6)	Bixler and D’Mello (2016)	First Pass Fixations	“*First fixation on each word during the first pass through the text*” (p.46)
	Mézière et al. (2025)	first-fixation duration	duration of first fixation on a word
	Nguyen et al. (2014) Exp 1 & 2	first-fixation duration	Not specified, word-level.
	Schad et al. (2012)	first-fixation duration	“*duration of the first fixation on a word (first-pass) irrespective of later eye movements*” (Supplementary Materials)
	Steindorf and Rummel (2020)	first-fixation duration	“*average first fixation […] for all words in one target sentence*” (p. 169)
Gaze Duration (*n* = 8)	Frank et al. (2015) YA^[2]^	gaze duration	“*the time from first fixation on a word until* *a* *fixation is made elsewhere*” (p.270)
	Mézière et al. (2025)	gaze duration	the sum of fixations made on a word during first-pass
	Nguyen et al. (2014) Exp 1 &2	gaze duration	Not specified
	Reichle et al. (2010)	gaze duration	“*the sum of all first-pass fixations on a word*” (p. 1304)
	Schad et al. (2012)	gaze duration	“*the cumulative duration of all first-pass fixations per word*” (p 184)
	Zhang et al. (2020) Exp 1 & 2	gaze duration	“*the sum of all fixations from entering the analysis region for the first* *time until leaving the region*” (p.4)
Total Reading Time (*n* = 7)	Mézière et al. (2025)	total reading time	sum of all fixations made on a word
	Nguyen et al. (2014) Exp 1 &2	total time	Not specified (word-level measure)
	Reichle et al. (2010)	total viewing time	*“the sum of all fixations on a word (including those occurring after interword regressions)”* (p.1304)
	Schad et al. (2012)	total reading time	“*the cumulative duration of all fixations on a word*” (Supplementary Materials)
	Zhang et al. (2020) Exp 1 & 2	total looking time	“*the sum of all fixations on the analysis region*” (p.4)
Fixation Count Word (*n* = 4)	Mézière et al. (2025)	fixation count word	number of fixations made on a word
	Nguyen et al. (2014) Exp 1 & 2	fixation count	word-level measure
	Schad et al. (2012)	number of passes	“*how often a word is read in total*” (Supplementary Material)
Interword Regressions (*n* =5)	Frank et al. (2015) YA & OA	interword regression	“*probability of regressing to the target word*”(p.55)
	Reichle et al. (2010)	interword regression count	*“saccades back to words that occurred earlier in the text”* (p.1303)
	Steindorf and Rummel (2020)	between-word regression count	“*number of observed occurrences of [regressions] during a given target sentence*” (p.169)
	Zhang et al. (2020) Exp 1 & 2	regressions-out	“*a* *count variable indicating the number of regressions from the analysis region to previous words*”

^2^For this measure, data was only available for the young adult sample

### Calculating effect sizes

**Fig. 2:**
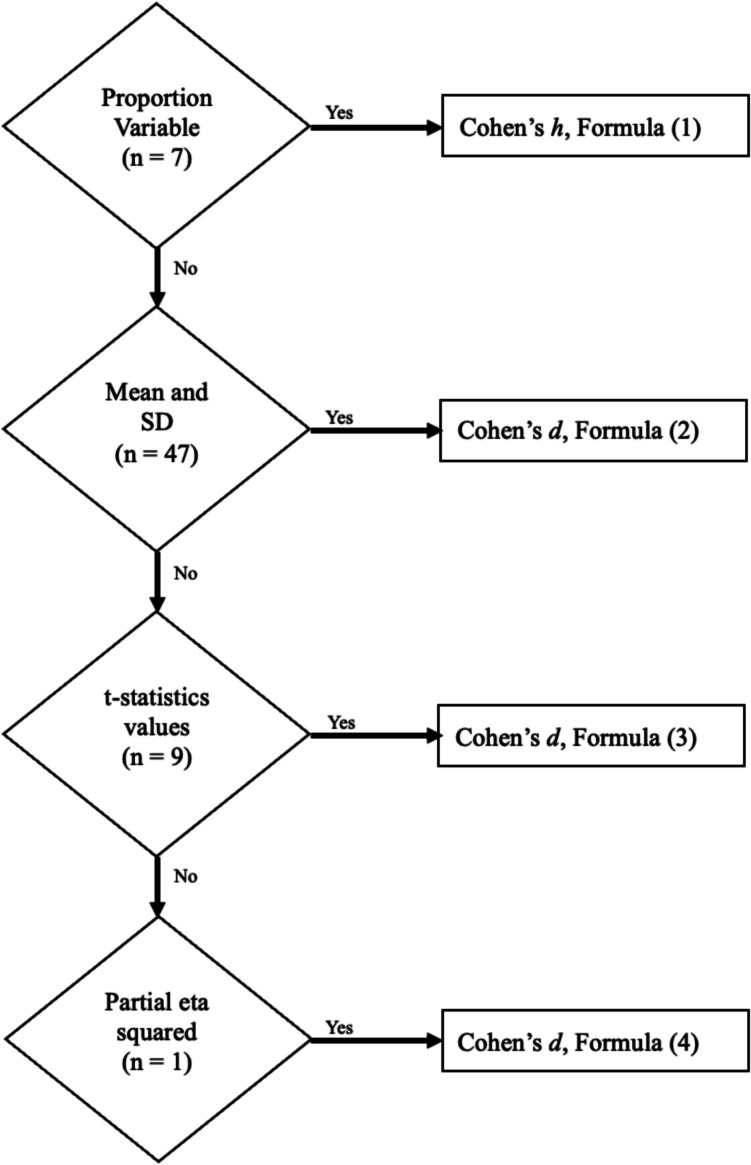
Decision tree for calculating effect sizes. The decision tree shows the sample sizes and formula used for computing the effect size for each eye-movement measure based on the type of measure and available information

The effect sizes were computed following the guidelines suggested by Cumming (2013). The decision tree and sample sizes for computing the effect sizes are shown in Fig. 2. For measures expressed as proportional values (i.e., skipping), we computed Cohen’s h (Cohen, 1977, Formula 6.2.2) using formula

1. Cohen’s h is an arcsine transformation of the difference in two proportions, used to describe the distance between two conditions P1 and P2, in which P1 is the proportion in the mind-wandering condition and P2 on-task. For other measures, if the mean and standard deviation were available, we calculated Cohen’s d using formula
2. Adapted from Cumming (2013, Formula 11.8).

In this formula, Cohen’s d is computed by taking the difference of the means of the two conditions, and standardizing it with the standard deviation of the on-task (task-related thoughts; TRT) condition, thus using it as the baseline. In cases where the article did not report sufficient data to compute the effect size this way, we used *t*-values, which we converted to an effect size with formula (3) following a methodology suggested by Borenstein et al. (2009, p. 228), in which the effect size is computed using the reported *t*-value and scaling it with a ratio of the sample sizes of two independent groups. For one paper, partial eta squared was reported, which we transformed into Cohen’s d using formula (4). Lastly, all effect sizes were further transformed to unbiased estimates of Cohen’s d (Cumming, 2013).

In instances where authors reported data for more than one analysis window (see Table 1), we calculated the average across time windows so as to only have one value per dataset. This was done in part because there was no standard window size across articles, such that no window size(s) could be chosen for comparison across datasets. Additionally, if there was more than one participant sample within the same study, we treated them as separate samples and calculated effect sizes for each sample separately. For example, the young adult and older adult samples in Frank et al. (2015) were treated as two separate datasets. This was done as research shows that younger and older adults differ in their eye-movement behavior and processing (see Zhang et al., 2022, for a meta-analysis) and hence they may have different results when it comes to mind wandering. Concerning one of the studies (Nguyen et al., 2014, Experiment 2), we only acknowledged the between-participant measures, since sufficient data for calculating the effect size were not available for within-participant measures. A handful of studies also allowed participants to report mind wandering during the experiments if they caught themselves mind wandering (*n* = 3), and thus made a distinction between prompt-caught and self-caught mind wandering in their analysis of eye-movement behavior. However, too few studies reported this distinction to calculate effect sizes for this analysis, hence we only considered prompt-caught episodes of mind wandering and did not include values for self-caught mind wandering. Across studies, mind wandering was defined as unintentional lapses of attention from the text, thus we could not include or compare results across different types of mind-wandering or thought patterns. The definitions of mind wandering used in each study are shown in Table 2.

**Table 2:** Descriptive Characteristics of Included Datasets

Authors	Year	Eye-Tracker			Participants			Stimuli		Measures	Window(s)	Mind-Wandering Definition
		Type	Sampling Rate	Gaze Algorithm	Type	N	Age	Length	Language			
Bixler and D'Mello	2014	Tobii TX300 OR Tobii T60	NA	Open Gaze & Mouse Analyzer	University students	178	20 (3.6)	4 x 1500 words	English	First-Fixation Duration Skipping Blink Count	4, 8, and 12 s pre-prompt variable, ends 2 s pre-prompt 4–10s pre-prompt 4,6, and 8 s pre-prompt 4, 6, 8, 10, 12 s to 3 s pre-prompt	"no idea what you just read" and "realizing you were thinking about something else altogether." “a decoupling between internal thoughts and processing of external stimuli; attention shifts from the current task to unrelated thoughts”
Bixler and D'Mello	2015											
Bixler and D'Mello	2016											
Bixler et al.	2015											
Faber et al.	2018											
Danckert et al.	2018	EyeLink II	250 Hz	NA	University students	25	19.56 (1.58)	Stories	English	Blink Count	10 s pre-prompt	Not clearly stated.
Faber et al.	2020	EyeLink 2k OR Tobii EyeX	1000 Hz 60 Hz	DataViewer Open Gaze & Mouse Analywer	University students	132	19.8 (1.51)	43 sentences	English	Fixation Durations Saccade Length Fixation Count (trial) Blink Count	15 s and 25 s pre-prompt	" found themselves thinking of something else altogether"
Foulsham et al.	2013	EyeLink II	500 Hz	Online algorithm: velocity (>30°/s) and acceleration (8000°/s)	University students	31	NA	48 sentences	English	Fixation Durations Saccade Length Reading Time Fixation Count (trial)	Whole sentence	task-unrelated thoughts draw resources away from the task; less attention to words being read
Frank et al.	2015	Applied Science Laboratories (model H6HS)	120 Hz	NA	University students Older adults	36 40	18–25 60–85	5 chapters	NA	Gaze Duration Skipping Regressions	3–8 s pre-prompt	
Gwizdka	2019	Tobii TX-300	NA	NA	NA	30	NA	3 passages	English	Fixation Durations Reading Time Fixation Count (trial)	5 s and 10 s pre-prompt	“reading did not result in any meaningful understanding of the text”
Méziere et al.	2025	EyeLink 1000	1000 Hz	Dataviewer (SR Research)	University students	56	23.43 (4.4)	Book chapters	Finnish	Fixation Durations Saccade Length Fixation Count (trial) Reading Time Blink Count First-Fixation Duration Gaze Duration Total Fixation Duration Fixation Count (word) Skipping	Whole paragraph pre-prompt	“mind-wandering, characterized by off-task thoughts whose content was unrelated to the narrative”
Nguyen et al. EXP1	2014	EyeLink 1000	1000 Hz	NA	Children K-2	27	NA	2 passages: -125 words -110 words	English	Saccade Length Fixation Count (word) First-Fixation Duration Gaze Duration Total Fixation Duration Skipping	Whole passage	“eyes moving across the page while the reader fails to fully process the text”
Nguyen et al. EXP2	2014	EyeLink 1000	1000 Hz	NA	Children K-5	135	NA	157 words	English			
Oyarzo et al.	2022	EyeLink 1000	500 Hz	NA	University students	40	21.6 (1.44)	– 67 000 words	Spanish	Fixation Durations Saccade Length Blink Count	5 s pre-prompt	“distracted and thinking about something else”
Reichle et al.	2010	EyeLink 1000	1000 Hz	NA	unspecified	4	NA	Whole book	English	Gaze Duration Total Fixation Duration Regressions	2.5s, 5 s, 10 s, 30 s, 60 s, and 120 s pre-prompt	“At some point during reading you realize that you have no idea what you just read and that not only were you not thinking about the text, you were thinking about something else altogether.”
Schad et al	2012	EyeLink 1000	500 Hz	SR Research Sofware	High-school students	30	17 – 20	50 stories with errors. 17 500 words total	German	Fixation Count (word) First-Fixation Duration Gaze Duration Total Fixation Duration Skipping	10, 14, and 20 words prior to error	Overlooking an error in text (lexical, syntactic, semantic, discourse or gibberish)
Smilek et al.	2010	EyeLink 1000	1000 Hz	NA	University students	15	NA	2 passages	English	Fixation Durations Fixation Count (trial) Blink Count	5 s pre-prompt	“thoughts completely unrelated to the text, such as thoughts about an upcoming meal (task-unrelated mind wandering)”
Steindorf & Rummel	2020	SMI RED500	500 Hz	BeGaze (SensoMotoric Instruments)	NA	122	22.58 (4.01)	58 pages	German	Fixation Count (trial) Blink Count Reading Time First-Fixation Duration Regressions	Whole sentence (target sentence pre-prompt)	“I am thinking about things unrelated to the text”
Zhang el al. - EXP1	2020	EyeLink 1000	500 Hz	NA	University students	47	18.96 (0.95)	Single sentences (garden-path jokes)	English	Gaze Duration Total Fixation Duration Skipping Regressions	Whole sentence pre-prompt	Unintentional mind wandering: "your thoughts drifted away despite your best intentions to focus on the task.”
Zhang el al. - EXP2	2020	EyeLink 1000	500 Hz	NA	University students	46	18.85 (0.89)	Single sentences (garden-path jokes)	English			

All analyses were carried out in R statistical software (R Core Team, 2023, version 4.3.1). Once we calculated effect sizes and standard errors for each measure, the summary effect sizes were computed with random-effects models using the DerSimonian-Laird method with the “*metaphor*” package (version 4.4.0, Viechtbauer, 2010). The forest plots were created with the “*metaviz*” package (version 0.3.1, Kossmeier et al., 2020). The analysis code and data are available at https://osf.io/v9dr2/.

1. $$h=2\left(\mathrm{arcsin}\sqrt{\left({P}_{1}\right)} -\mathrm{arcsin}\sqrt{\left({P}_{2}\right)} \right)$$
2. $$\mathrm{d}=\frac{\overline{{\mathrm{y} }_{\mathrm{trt}}} - \overline{{\mathrm{y} }_{\mathrm{mw}}}}{{\mathrm{s}}_{\mathrm{trt}}}$$
3. $$\mathrm{d}=\mathrm{t}\sqrt{\frac{{\mathrm{n}}_{1}+{\mathrm{n}}_{2}}{{\mathrm{n}}_{1}{\mathrm{n}}_{2}}}$$
4. $$\mathrm{d}=\sqrt{\frac{\left(\mathrm{n}-1\right){\upeta }_{\mathrm{p}}^{2}}{\mathrm{n}/\left(1-{\upeta }_{\mathrm{p}}^{2}\right)}}$$

### Results

#### Overview of the included studies

Characteristics of the studies for which data were extracted are summarized in Table 2. Most of the studies were conducted in English (*n* = 11), two were conducted in German, one in Spanish, and one in Finnish. Most studies were conducted with university students (*n* = 10), one included older adults, and two were conducted with school-aged children. Most experiments used passages of text as the stimuli (*n* = 12) from existing works of fiction (e.g., “*War and Peace*” by Tolstoi) or non-fiction (e.g., “*Musicophilia: Tales of Music and the Brain*” by Oliver Sacks), although the length of the passages varied greatly from ~ 150 words to the whole of Jane Austen’s “*Sense and Sensibility.*” Only four datasets used single sentences as stimuli, which typically also included some experimental manipulations such as word frequency or garden-path structures.

Articles included for data extraction typically fell into two main categories: experiments investigating the influence of mind wandering on eye-movement behavior during reading (*n* = 14), and machine-learning studies focusing on predicting mind-wandering episodes from eye-movement features (*n* = 6; although five of these used the same dataset). As a result, the analysis methods varied across studies. Machine-learning studies primarily tested the usefulness of eye-tracking measures and machine-learning algorithms to predict mind wandering during reading, while other studies investigated the effect of mind wandering on eye movements. This difference in aims also led to differences in the type and number of eye-movement features used and reported across studies. Indeed, machine-learning studies typically extracted more eye-movement measures (e.g., 66 features of eye-movement behavior were extracted in Bixler and D’Mello, 2015), and tended to include measures that are less commonly used in eye-tracking research and which do not have a common interpretation as to cognitive processing in the literature (e.g., kurtosis of fixation durations). The window of analysis also varied greatly across studies, and fell into two categories such that eye-tracking measures were collected and aggregated either over the whole trial/sentence (*n* = 7), or only for a specific region of interest/time window (*n* = 8). For articles in which a time window was used, the size of the window varied both within and across studies such that there was no standard time window used to examine eye-movement behavior during mind wandering.

Most of the studies collected eye-movement data with EyeLink eye trackers (*n* = 12) or Tobii eye trackers (*n* = 3), which are the most commonly used eye trackers in reading research. The eye trackers were set up with a sampling rate of at least 500 Hz in most studies (*n* = 12), although studies using Tobii eye trackers tended to have lower sampling rates (< 500). Few studies reported which algorithm was used to detect saccades and fixations from the raw eye-movement data, although it is likely that studies using EyeLink eye trackers used the SR Research software DataViewer. As pointed out by a reviewer, such differences between studies in sampling rate, algorithm for saccade and fixation extraction, as well as pre-processing of the data are likely to impact data quality. In the sample of studies included in the meta-analysis, however, we did not find any evidence that studies using a particular eye tracker (e.g., EyeLink vs. Tobii) or sampling rate (over or below 500 Hz) were more likely to show significant effects or effects in a particular direction.

Across studies, mind wandering was typically defined as “thinking about something other than the text.” While a handful of studies did investigate other types of mind wandering, there were not enough data across datasets for us to include them. Hence, we only included data from mind-wandering episodes defined as unintentional task-unrelated thoughts (see *Limitations*). Nearly all studies identified mind-wandering episodes by prompting participants at regular or semi-regular intervals during reading and asking them to report whether they were on-task or not (*n* = 14). One exception was the study by Nguyen et al. (2014), in which mind wandering was identified by expert raters based on visualization of eye-movement patterns during reading.

#### Eye-movement markers of mind wandering

**Fig. 3:**
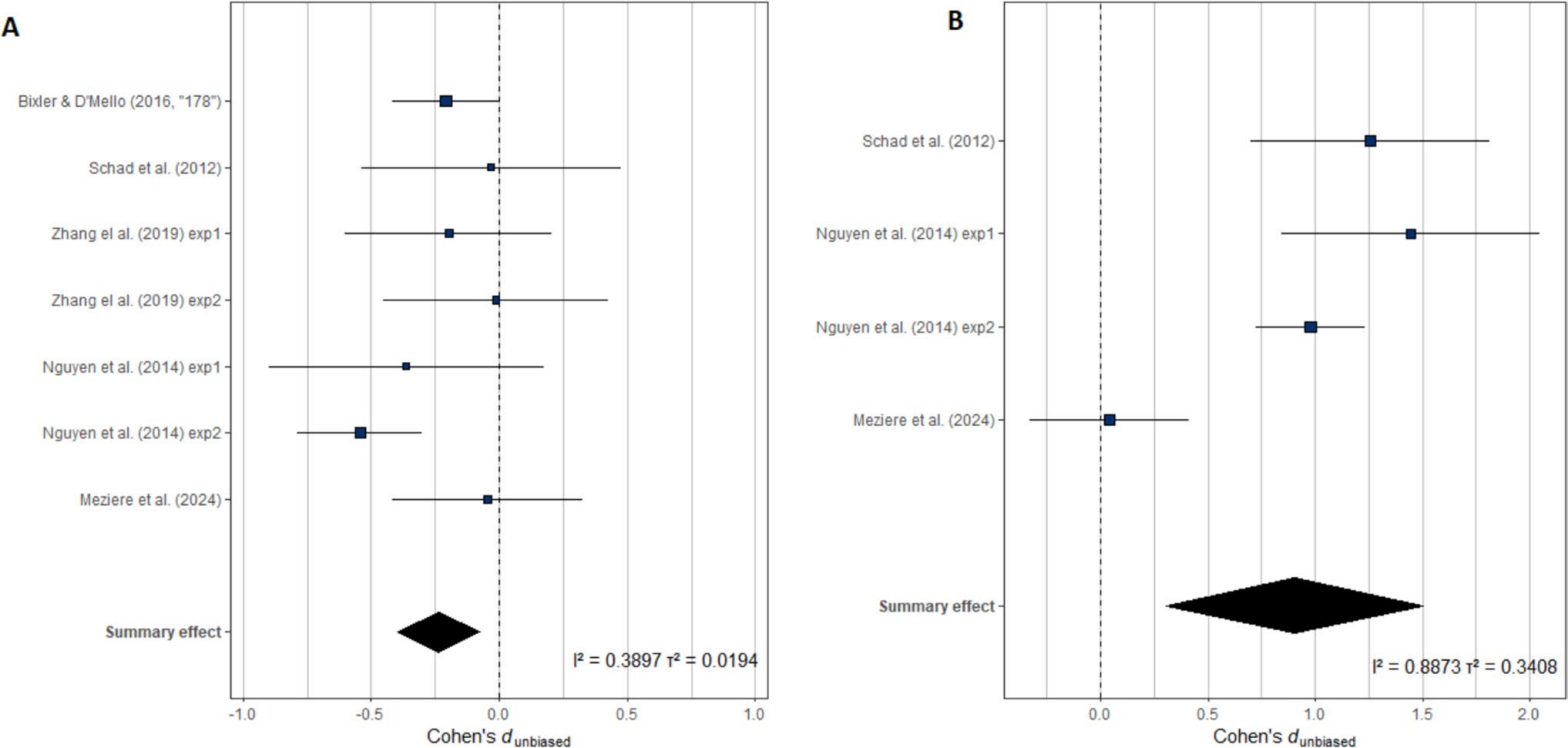
Summary of effect sizes for significant measures. Summary of effect sizes per dataset for skipping rate (**A**) and number of fixations per word (**B**). Positive effect sizes indicate higher values during on-task reading, and negative values indicate higher values during mind wandering. Heterogeneity indexes *I*^*2*^ and *Τ*^*2*^ are also included

The summary effect sizes and confidence intervals for each measure are shown in Table 3. Results of the meta-analysis indicated that only two eye-movement measures were significantly impacted by mind wandering compared to on-task reading. Of our six global measures, only skipping rate was significantly different between mind-wandering episodes and on-task reading, such that readers skipped more words during mind wandering (*d* = −0.24, 95% CI: −0.40 to −0.07). The *I*^2^ heterogeneity index in this model was 39%, suggesting low to moderate heterogeneity. Of our five word-level measures, only number of fixations per word was significantly related to mind wandering (*d* = 0.90, 95% CI: 0.30–1.50), as readers made fewer fixations per word during mind-wandering compared to on-task reading. The *I*^2^ heterogeneity index in this model was 89%, suggesting high heterogeneity. These effects are illustrated in Fig. 3.

**Table 3:** Effect sizes and confidence intervals for each eye-movement measure

Measure	Cohen’s d (unbiased)	95% confidence interval
Mean fixation duration (*n* = 6)	−0.41	[−0.92, 0.11]
Mean saccade length (*n* = 6)	0.24	[−0.11, 0.60]
Skipping rate (*n* = 7)	**−0.24**	**[−0.40, −0.07]**
Blink count (*n* = 7)	0.01	[−0.21, 0.23]
Trial-level fixation count (*n* = 6)	0.01	[−0.22, 0.23]
Trial-level total reading time (*n* = 3)	−0.23	[−0.52, 0.06]
First-fixation duration (*n* = 6)	−0.10	[−0.27, 0.08]
Gaze duration (*n* = 8)	0.02	[−0.12, 0.16]
Total reading time (*n* = 7)	0.07	[−0.10, 0.24]
Fixation count (*n* = 4)	**0.90**	**[0.30, 1.50]**
Inter-word regression count (*n* = 5)	−0.15	[−0.46, 0.16]

Positive effect sizes indicate higher values during on-task reading, and negative values indicate higher values during mind wandering. Bold font indicates that the 95% confidence interval does not include 0

In addition to the studies included in the effect-size calculations, we identified six studies that fulfilled the inclusion criteria but which did not report the necessary information to calculate the effect-size estimates and for which we did not get access to the data (Bixler & D’Mello, 2021; Brishtel et al., 2020; D’Mello et al., 2016, 2017; Hutt et al., 2024; Uzzaman & Joordens, 2011). While the data from these papers could not be included in the meta-analysis, we review their findings here as to possible indicators of mind wandering that may not have been identified in the meta-analysis. Uzzaman and Joordens (2011) compared eye-movement measures during mind-wandering and on-task thought, and found that the number of within-word regressions and number of visits to a word were reduced during mind wandering. Moreover, the number of fixations and saccades was lower during mind-wandering episodes than during on-task reading, even though these effects did not reach statistical significance. Hutt et al. (2024) used a webcam-based eye tracker and applied a set of different classifiers to predict mind-wandering episodes on the basis of the eye-movement data, and showed that a combination of local (i.e., AOI-based measures) and global (i.e., number of gazes and their dispersion on the screen) measures was the best predictor of mind-wandering episodes. However, they did not report which measures ranked the best. In contrast, in studies reported by D’Mello and colleagues (2016, 2017), the best models only included global eye-movement features. Bixler and D’Mello (2021) and Brishtel et al. (2020) used machine learning to predict mind wandering during reading, but they only reported the best eye-movement features that predict mind wandering. In the study by Bixler and D’Mello (2021), these features were related to saccade velocity, duration, amplitude, and angle. Median fixation duration and blink count were also among the best predictive features. Brishtel et al. (2020) also found that parameters related to saccade velocity and angle and maximum fixation duration were among the best predictors of mind wandering. In addition, they reported that the number of regressions, mean regression length, and parameters related to pupil size were predictors of mind wandering. Even though the results of these studies seem variable, they do suggest that saccadic activity, and possibly regressive eye movements, differentiate between mind-wandering and on-task reading.

#### Publication bias and selective reporting

**Fig. 4:**
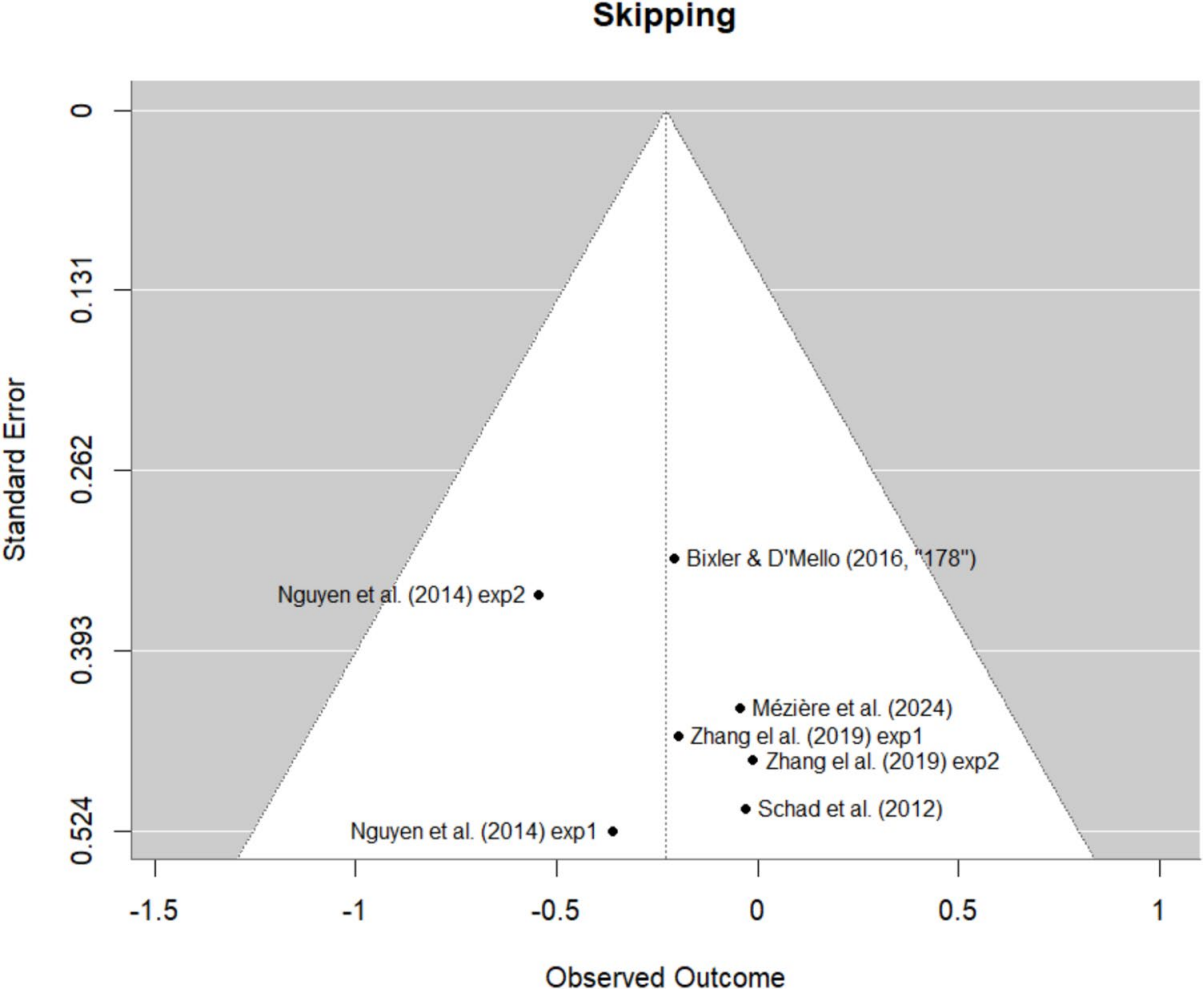
Funnel plots for skipping rates

**Fig. 5:**
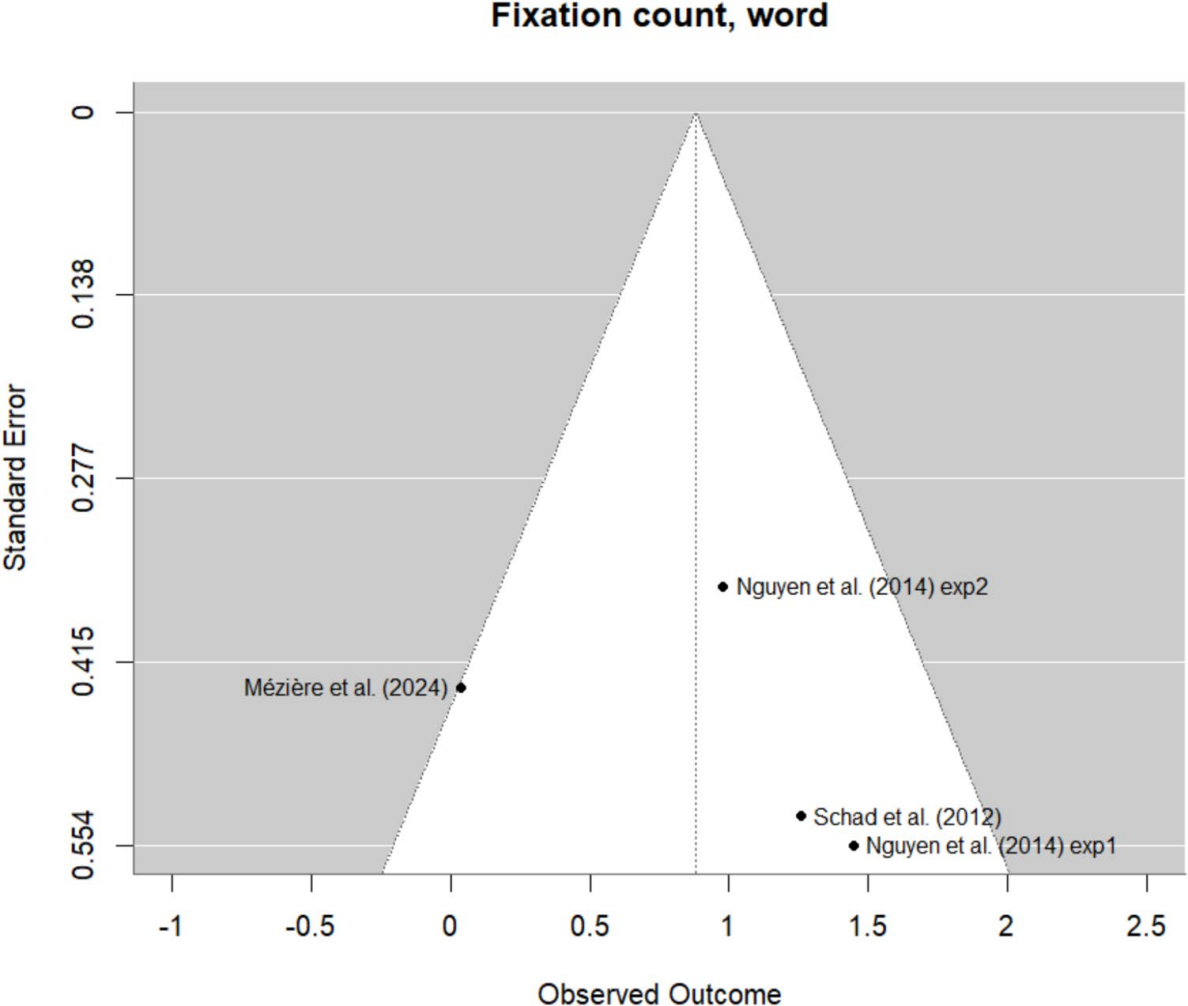
Funnel plot for word-level fixation numbers

Possible publication bias was investigated by visualizing funnel plots for each eye-tracking measure reported in the meta-analysis using the “*metafor*” package (version 4.4.0, Viechtbauer, 2010). The funnel plots for skipping rates and word-level fixation number are shown in Figs. 4 and 5 (see Online Supplementary Material for funnel plots for all measures). The funnel plots do not suggest publication biases for either measure. However, the number of studies is rather limited, especially for word-level fixation counts, hence clear signs of publication bias may be difficult to identify.

### Discussion

The results of this meta-analysis suggest that mind wandering, when defined as instances of “unintentional task-unrelated thoughts,” is associated with higher overall skipping rates and fewer fixations per word. While these results might be expected to go hand in hand with faster reading speed due to fewer words being fixated during reading and fewer fixations on individual words, such reduced reading times were not found for any of the global or local fixation duration measures included in the analysis. On the contrary, we found small to medium effect sizes for mean fixation durations (*d* = −0.41), mean saccade length (*d =* 0.24), and total trial reading times (*d* = −0.23), suggesting that mind wandering may be associated with longer overall reading times as well as longer fixations and shorter saccades. This would be in line with previous findings suggesting that mind wandering might be predicted by longer fixations and overall reading times (e.g., Bixler & D’Mello, 2016; Bixler et al., 2015; Faber et al., 2018). However, these effects were not significant in this meta-analysis, which may be due in part to the small number of studies available for the analysis, leading to large confidence intervals. Indeed, an important caveat to the results of the meta-analysis is that for most of the measures we included the number of datapoints was very limited. While we identified 16 datasets we could include in the analysis, the most commonly reported measure across datasets (i.e., gaze duration) was only reported (or available) for eight (i.e., 50%), and for the less commonly reported measures only three (i.e., 18.75%) datapoints were available. Hence, the lack of significant effects may be due, at least in some cases, to the low number of datapoints available for our analysis (see *Limitations*).

Results from the meta-analysis suggest that word-skipping behavior and the number of fixations per word are useful indicators of mind wandering during reading. In the next sections, we report results from additional exploratory analyses in which we aimed to further understand these results by examining skipping behavior during mind wandering compared to on-task reading.

#### Limitations

An important limitation of the meta-analysis presented in this article comes from the great variability across articles in their methods, particularly in the number and types of eye-tracking measures they reported, ranging from 1 (Danckert et al., 2018) to 80 eye-tracking measures (Bixler & D’Mello, 2016). Indeed, as noted in earlier sections, there is no standard as to which eye-movement features were reported or examined across studies. Hence, while a plethora of measures were considered as indicators of mind wandering across all the studies reviewed in our meta-analysis, there were only 11 measures which were reported in at least three of the articles and for which we could calculate effect sizes. Thus, the number of available data points across measures was quite low, ranging from eight (gaze duration) to only three (trial-level total reading time). It may therefore be that for some of the measures included in our analysis we did not have enough data points to reliably estimate the effect size. Nevertheless, while these numbers are low, recent discussion on statistical power for running meta-analysis argues that as few as two studies are enough to run a meta-analysis (Valentine et al., 2010). In addition, the measures reported in the meta-analysis are only a small subset of all the measures reported across studies, and hence do not constitute an exhaustive list of possible candidates for useful indicators of mind wandering during reading. For example, as noted earlier, we were not able to include measures of dispersion and shape of the distribution in eye-movement behavior, or measures of sensitivity to lexical properties of text, although these types of measures are good candidates for eye-movement markers of mind wandering during reading. Hence, while we did identify two potential indicators of mind wandering, more research looking at other types of eye-movement measures is necessary to examine other useful predictors of mindless reading.

Another important aspect in which studies varied was the analysis window or area of interest on which the analysis was carried out. We found that there is no standard in the literature as to the size of the window of analysis to compare mind-wandering episodes to on-task reading. An important empirical question that arises is therefore whether the size of the chosen analysis window has an impact on the results, and how long before the probe readers were mind wandering. Indeed, only a few articles considered multiple windows of analysis, and typically found that the results could vary across analysis windows (e.g., Faber et al., 2020; Reichle et al., 2010). However, given the variability of the window of analysis across studies, we were not able to examine this possible influence of the window size on the results, hence we cannot be sure that the results of the meta-analysis generalize across analysis windows.

Lastly, as noted in earlier sections, this article focused on research on mindless reading in which mind wandering was defined as “involuntary task-unrelated thoughts,” as this definition of mind wandering was commonly used across the articles that came up in our search. Hence, an important caveat of the results of the meta-analysis is that they may not generalize to other types of mind wandering. Indeed, only a few studies included other types of mind wandering in their design and thus investigated not only the contrast between on-task and off-task reading but also considered instances of self-caught mind wandering (instances where the reader detects they are mind wandering without being probed; Oyarzo et al., 2022; Reichle et al., 2010), task-related interruptions (i.e., thoughts related to the text: Frank et al., 2015; Steindorf & Rummel 2020), or intentional mind wandering (Zhang et al., 2020). Importantly, findings across these studies do suggest that eye-movement behavior does differ not only between on-task and off-task reading but also between subtypes of mind wandering, thus suggesting that the results of our meta-analysis cannot be taken to generalize to other types of mind wandering. Therefore, these differences across types of mind-wandering episodes call for further research into the different types of mind wandering that occur during reading, and how they affect the reading process including eye-movement behavior, cognition, and comprehension outcomes.

## Study 2: Exploratory analyses

Following the results of the meta-analysis, we ran further exploratory analyses in order to shed light on plausible interpretations of the effect of mind wandering on eye-movement behavior. Specifically, we considered two plausible explanations for the finding that mind wandering was associated with higher skipping rates and fewer fixations compared to on-task reading by investigating possible effects of (1) characteristics of the words that were or were not skipped, and (2) *when* readers did and did not fixate words.

First, we considered possible differences in the effects of word length and word frequency on skipping rates during mind wandering compared to on-task reading. Indeed, a plausible explanation for higher skipping rates and fewer fixations during mind wandering may be due to readers being less sensitive to linguistics variables of the text, as previous findings suggest that readers show a reduced word-frequency effect during mind-wandering compared to on-task reading (Reichle et al., 2010; Smilek et al., 2010). Hence, it may be that, during mind wandering, readers are more likely to skip infrequent and long words as they may make less use of visual (i.e., word length) and linguistic (i.e., word frequency) information compared to on-task reading. This would be in line with the predictions of the *cascade model of inattention* proposed by Smallwood (2011), which posits that during mind wandering readers’ lexical processing may be less detailed compared to on-task reading, leading to a reduced sensitivity to word characteristics such as word frequency.

Secondly, we considered *when* a word was skipped. Indeed, the skipping rates included in the meta-analysis were whole-text skipping rates, namely the proportion of words that were *never* fixated during reading. This measure contrasts with first-pass skipping rates, which indicate words that were skipped when they were first encountered, although they may have been fixated later on during reading. This distinction is important, as words may be skipped during first pass due to several reasons, such as (1) they are highly frequent, predictable, and/or short words (Brysbaert & Vitu, 1998; Driegue et al., 2004); (2) readers were able to identify the word from parafoveal preview while fixating the previous word (e.g., Blanchard et al., 1989); (3) oculo-motor error due to overshooting (McConkie et al., 1988, 1994). Hence, while successful word identification has typically occurred in the first two cases, the latter case is often followed by a corrective regression in which the reader returns to the skipped word in order to identify it, as studies show that skipped words tend to receive such immediate regressions much more often than non-skipped words (Drieghe et al., 2004; Vitu & McConkie, 2000). A plausible explanation for higher overall skipping rates during mind wandering could therefore be that readers are less likely to make such corrective regressions during mind-wandering compared to on-task reading.

In these exploratory analyses, we tested two hypotheses:

1. Readers will show reduced effects of word frequency and word length on skipping rates during mind-wandering compared to on-task reading.
2. Readers will be less likely to make corrective regressions to a word skipped in first pass during mind-wandering compared to on-task reading, particularly if the words are long.

### Methods

#### The dataset

We ran the exploratory analyses with a dataset collected in our own lab. This dataset was collected for a study reported in Mézière et al., 2025 which explored the relationship between eye-movement behavior during reading and immersion. For convenience, we report the key details here.

#### Participants

The dataset contains eye-movement data from 56 native Finnish speakers (51 women, 52 right-handed, mean age: 23.43 years).

#### Stimuli

Participants read passages from Siri Hustvedt’s novel “Memories of the Future” (Hustvedt, 2019). Participants could read the text at their own pace and pressed the keyboard to move to the next paragraph. The paragraphs were short, ranging from 27 to 151 words, and participants spend on average 30 s reading each paragraph (standard deviation ~14 s). The prompts occurred after 30 target paragraphs at intervals of around 5 min between prompts, and the whole experiment took around 2 h to complete.

#### Eye-movement data

Participants’ eye movements were collected with an EyeLink 1000 system (SR Research Ltd.) at a 1,000-Hz sampling rate. Eye-movement features (i.e., fixations and saccades) were extracted using the DataViewer software from SR Research. The text was presented one paragraph at a time on a computer screen using 15-point LucidaConsole and triple line spaces. Eye-movement data were collected for the entire time participants were reading the paragraphs.

#### Mind-wandering data

Participants were prompted after 30 target paragraphs using a 13-item Multidimensional Experience Sampling (mDES) questionnaire (Turnbull et al., 2019). The first item of this questionnaire asked participants to answer on a scale from 1 to 4 whether they were on task (i.e., 4) or not (i.e., 1). As only mind wandering was of interest in the current article, we did not consider answers on any of the other 12 items of the questionnaire. Mind wandering was defined as answers 1–2 on item 1, and on-task reading as answers 3–4. The dataset is available at https://osf.io/kd8xa/.

#### Procedure

The experiment took place in a quiet laboratory at the University of Turku. Participants signed an informed consent prior to participating in the experiment. They were told that they would be reading passages from a novel, and to read at their own pace, as they do when reading for pleasure. They were then familiarized with the mDES questionnaire, and each item was explained to them. The eye tracker was then calibrated using a 9-point calibration scheme, and participants completed a short example trial to familiarize themselves with the task. Each trial then started with a drift check and participants could start reading by pressing the space bar and moved to the next trial by pressing the space bar again.

#### Data analysis

The eye-movement data were cleaned using the “*PopEye*” package in R (Schroeder, 2019). All analyses were conducted in R (R Core Team, 2023). Linear models were run using the “*lme4*” package (Bates et al., 2015) and visualizations of results were produced using the “*ggplot2*” package (Wickam, 2016), “*sjPlot*” package (Lüdecke, 2024), and “*patchwork”* package (Pedersen, 2024). Post hoc comparisons were run with the “*emmeans*” package (Lenth, 2024).

Firstly, we examined the effects of word length and word frequency on skipping behavior during mind-wandering and on-task reading. To do this, we ran linear mixed models with skipping rates as our outcome variable, with mind wandering, word length, or word frequency and their interaction as predictors. Word frequency was log-transformed prior to running the model to meet the normality assumption of the model. As word length and word frequency are highly correlated (*r* = −0.73), we ran separate models for each variable. Whenever possible (i.e., model convergence), we also included random intercepts and random slopes for participants and items as random factors. To further examine differences between mind-wandering and on-task reading at different levels of word frequency and word length we ran post hoc comparisons.

Secondly, we examined the effect of mind wandering on the proportion of corrective regressions (i.e., regressions following a word skipped during first pass) made during reading. In this analysis, we only considered words that had been skipped during first-pass reading. We investigated the probability of making a corrective regression in two ways. Firstly, we considered the number of words which were never fixated after being skipped during first pass, using overall skipping rates as our outcome variable. Secondly, we looked at regressions made into a word during first-pass reading which had previously been skipped. To control for the effects of word length on skipping rates, we included word length and its interaction with mind wandering as predictors in this model. As word frequency and word length were highly correlated, we did not control for word frequency on top of controlling for word length. To examine whether the effects of mind wandering on corrective saccades at different levels of word length we also ran post hoc comparisons.

### Results

#### Word length and frequency effects on skipping rates

**Fig. 6:**
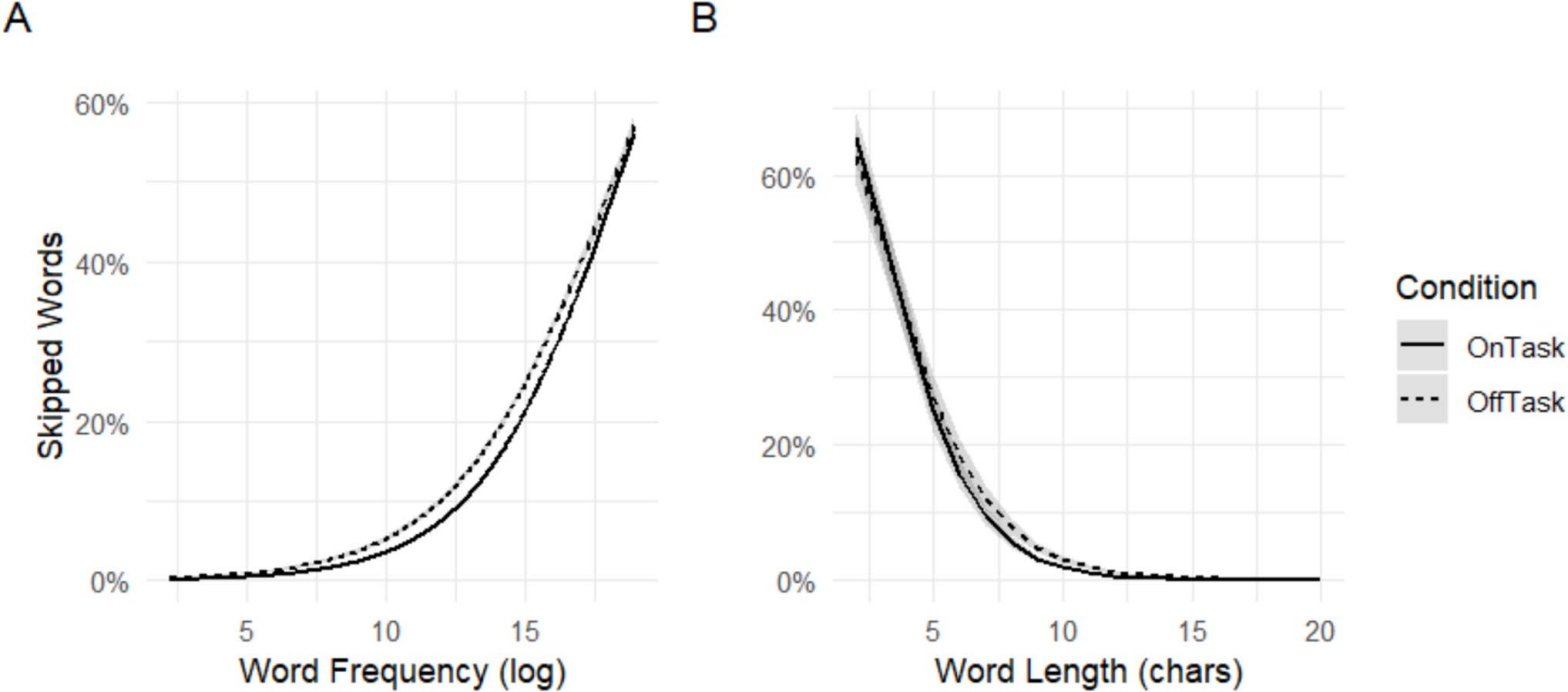
Effects of word frequency (**A**) and word length (**B**) on skipping rates as a function of participants’ being on-task or off-task

Our first exploratory analysis examined the effect of word length and word frequency on skipping rates during mind-wandering and on-task reading. The output of these models is shown in Table 4. Results showed main effects of mind wandering (*b* = 0.80, CI: = 0.60–1.01, *p* < 0.001), word frequency (*b* = 0.40, CI = 0.39–0.41, *p* < 0.001), as well as a significant interaction between the two (*b* = −0.04, CI = −0.05 to −0.03, *p* < 0.001). The interaction showed that the effect of word frequency on skipping rates was smaller during mind-wandering compared to on-task reading. Post hoc comparisons are shown in Table 5 and show that differences in skipping rates between mind-wandering and on-task reading were significant for low- (log frequency = 5), medium- (log frequency = 10), and high-frequency (log frequency = 15) words but that this difference was largest for low- and medium-frequency words. Similarly, results from the word length models showed significant main effects of mind wandering (*b* = −0.30, CI = −0.38 to −0.21, *p* < 0.001), word length (*b* = −0.58, CI = −58 to −0.56, *p* < 0.001), and a significant interaction (*b* = 0.08, CI = 0.06–0.09, *p* < 0.001), such that the effect of word length was smaller during mind-wandering than during on-task reading. These interactions are illustrated in Fig. 6, showing that while readers did skip short and frequent words more often than long and infrequent words in both conditions, they did so to a lesser extent during mind wandering. The post hoc comparisons shown in Table 6 are consistent with this and show that skipping rates were lower during mind wandering compared to on-task reading for short (five characters), medium (ten characters), and long words (15 characters), but that this difference was largest for medium and long words.

**Table 4:** Output of word frequency and word length models

Predictor	Word frequency		Word length	
	*b*	95% CI	*b*	95% CI
Intercept	−7.32*	−7.43 to - 7.21	1.77*	1.60–1.94
Mind wandering	0.80*	0.60–1.01	−0.30*	−0.38 to −0. 21
Word frequency	0.40*	0.39–0.41		
Word frequency * Mind wandering	−0.04*	−0.05 to −0.03		
Word length			−0.57*	−0.58 to −0.56
Word length * Mind wandering			0.08*	0.06–0.09

Output of the models for word frequency and word length on skipping rates

* = *p* < 0.05

**Table 5:** Pairwise comparison in skipping rates between on-task reading and mind wandering for low-frequency (log frequency of 5), medium-frequency (log frequency of 10), and high-frequency words (log frequency of 15)

	Low-frequency words				Medium-frequency words					High-frequency words				
	B	SE	z ratio	*P*		b	SE	z ratio	*P*		b	SE	z ratio	*P*
Mind wandering	−0.56	0.09	−6.59	<.001		−0.37	0.06	−6.22	<.001		0.18	0.05	−3.93	<.001

**Table 6:** Effects of mind wandering on skipping rates per levels of word length

	Short words				Medium words					Long words				
	b	SE	z ratio	*P*		b	SE	z ratio	*P*		b	SE	z ratio	*P*
Mind-Wandering	−0.09	0.02	−4.51	<.001		−0.48	0.05	−10.39	<.001		−0.86	0.08	−10.28	<.001

#### Corrective regressions

**Fig. 7:**
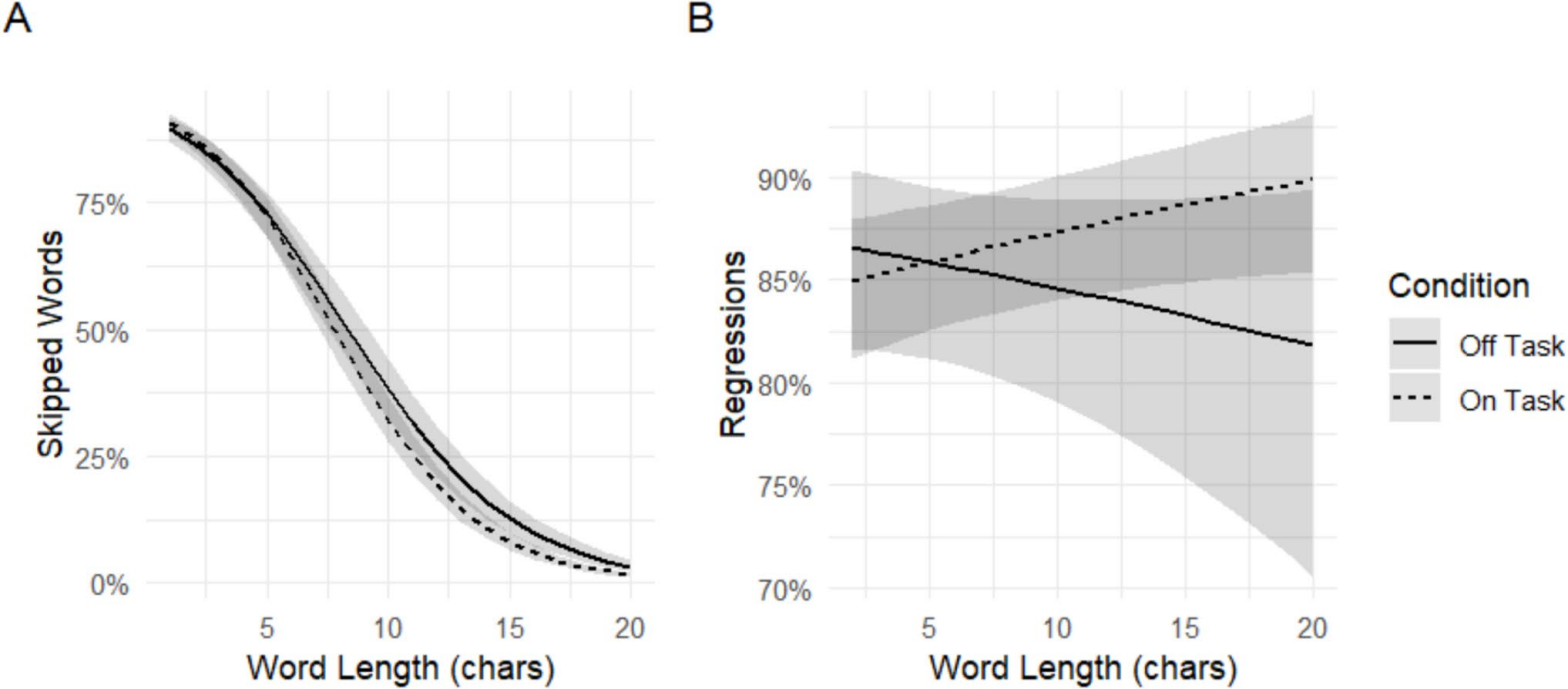
The proportions of words that were never fixated (**A**) and regressed to during first-pass (**B**) after they were skipped during first pass, as a function of participants’ being either on task or off task

In our second exploratory analysis, we examined the probability of fixating a word that was skipped during first-pass reading (i.e., a corrective regression). The output of these models is shown in Table 7. The model with full skipping rates as the outcome variable showed main effects of mind wandering (*b* = 0.20, CI = 2.20–2.68, *p* = 0.006), word length (*b* = −0.29, CI = −0.31 to −0.27, *p* < 0.001), as well as a significant interaction (*b* = −0.05, CI = −0.07 to −0.03, *p* < 0.001), showing that readers were less likely to fixate a word they had skipped during first-pass reading when they were mind wandering, especially for long words. Pairwise comparisons shown in Table 8 are in line with this, showing that differences in skipping rates between mind-wandering and on-task reading were only significant for medium and long words (10 and 15 characters, respectively). The model with first-pass regression probability as the outcome variable only showed a significant interaction between word length and mind wandering (*b* = 0.05, *p* = 0.05), such that readers were less likely to make a regression into a word when they were mind wandering and the word was long. These interactions are illustrated in Fig. 7. Pairwise comparisons shown in Table 9 are in line with this, showing that the differences in the probabilities of making a regression into a word that was skipped during first-pass reading were significantly lower during mind wandering only for long words (15 characters).

**Table 7:** Output of the corrective regression models

Predictor	Words skipped		Regressions	
	*B*	95% CI	*b*	95% CI
Intercept	2.44*	2.20–2.68	1.95*	1.57–2.33
Mind wandering	0.20*	0.06–0.34	−0.30	−0.64–0.05
Word length	−0.29*	−0.31 to −0.27	−0.03	−0.07–0.01
Mind wandering * Word length	−0.05*	−0.07 to −0.03	0.05*	0.01–0.10

*Note.* Output of the models on the probability making corrective regressions after a word was skipped during first pass

* = *p* < 0.05

**Table 8:** The output of post hoc comparisons in corrective regressions (total words skipped from words that were skipped during first-pass reading) for short (five characters), medium (ten characters), and long words (15 characters)

	Short words				Medium words				Long words			
	b	SE	z ratio	*p*	b	SE	z ratio	*p*	b	SE	z ratio	*p*
Mind wandering	0.04	0.04	0.92	0.356	0.28	0.07	3.94	<0.001	0.51	0.12	4.26	<0.001

**Table 9:** The output of post hoc comparisons in corrective regressions (probability of making a regression into a word that was skipped during first-pass reading) for short (five characters), medium (ten characters), and long words (15 characters)

	Short words				Medium words				Long words			
	B	SE	z ratio	*p*	b	SE	z ratio	*p*	b	SE	z ratio	*p*
Mind wandering	0.02	0.12	0.19	0.85	−0.25	0.15	−1.66	0.097	−0.52	0.23	−2.23	0.03

### Discussion

The aim of the exploratory analyses was to examine plausible explanations for the results of the meta-analysis. Specifically, we tested two hypotheses.

Our first hypothesis was that readers would show reduced effects of word frequency and word length during mind wandering compared to on-task reading. Our results showed that the size of both the word-length and the word-frequency effects were indeed reduced during mind-wandering episodes compared to on-task reading. These results are in line with previous studies showing reduced effects of word frequency and word length on fixation-duration measures (Foulsham et al., 2013; Reichle et al., 2010; Schad et al., 2012; Steindorf & Rummel, 2020; Uzzaman & Joordens, 2011) such that while readers did fixate long/infrequent words longer than short/frequent words, this difference was significantly reduced and even non-significant in short intervals before readers indicated that they had been mind wandering (e.g., 2.5–10 s prior to the prompt; Reichle et al., 2010). These results suggest that lexical processing differs between on-task and off-task reading, as readers seem to be less sensitive to lexical properties of words during mindless reading. This is in line with Smallwood’s (2011) cascade model of inattention which proposes that when attention is decoupled from the text, lexical processing will be less detailed compared to on-task reading, which would lead to poorer mental representations of the text and in turn poorer comprehension. Taken together, these results suggest that readers’ sensitivity to lexical properties of the text may be a useful indicator of mind-wandering episodes during reading.

Our second hypothesis was that the higher skipping rates during mindless reading compared to on-task reading may be due to readers making fewer corrective regressions (i.e., regressions back to a word that was accidentally skipped) during mindless reading. Our results showed that readers were indeed less likely to make a regression back to a word that they had skipped during first pass when they were mind wandering compared to on-task reading. This effect was only apparent for longer words, however, as very short words (< five characters) were skipped over 75% of the time during both on-task and off-task reading. This suggests that the higher skipping rates in mindless reading may be due (at least in part) to readers not correcting for oculomotor errors such as overshooting, and thus skipping a word that they meant to fixate and process. This is also in line with the view that, during mindless reading, readers’ lack of attention to the text may lead them to not notice that a word was accidentally skipped and hence not correct the error. These results also suggest that while measures of central tendency such as average overall skipping rates may be useful indicators of mind wandering, measures indicative of *when* a word is skipped may also be a useful (and perhaps more discriminating) marker of mind wandering.

#### Limitations

The limitations of the additional analyses presented here are similar to those discussed with regards to the meta-analysis. Indeed, the dataset included eye-movement data collected while participants read the entire paragraph that preceded the prompt, hence we cannot generalize the results to other windows of analysis including longer or shorter time windows prior to the prompt. Indeed, in our analysis we aggregated data across analysis windows for studies that reported results for more than one window. Although this was done because no single window could be chosen for the analysis, it is an important limitation as it is likely that the results would be impacted by the size of the analysis window. In addition, the design of the experiment did not allow for participants to report instances of mind wandering at times other than the prompt, so we could not examine whether the results of our analyses replicated in instances where readers caught themselves mind wandering without being prompted (e.g., Reichle et al., 2010). Similarly, the mind-wandering data used in these analyses defined mind wandering as being “off-task” (i.e., attention was not focused on the reading task) and did not examine type or contents of the thoughts in more detail, hence the results of these analyses may not generalize to other types of mind wandering such as intentional mind wandering (see Seli et al., 2016, 2018). The contents of the thoughts during mind wandering may also vary on several dimensions (see, e.g., Ruby et al., 2013). Such distinctions are important as research suggests that different types of thought patterns are reflected differently in eye movements (e.g., Mézière et al., 2025).

## General discussion

The primary aim of this article was to identify possible eye-movement indicators of mind wandering (i.e., task-unrelated thoughts) during reading. Taken together, the results of the meta-analysis and the exploratory analyses suggest that while some measures of central tendency in eye-movement behavior such as overall skipping rates and word-level fixation counts are useful indicators of mind wandering, measures that more directly indicate differences in cognitive processing may also be useful indicators of mindless reading. For example, the results suggest that a reduced (or non-existent) sensitivity to lexical properties of words such as word frequency or length can be indicative of mindless reading as characterized by less detailed lexical processing compared to on-task reading. Similarly, while skipping rates may be useful indicators of mind wandering, measures that take into account *when* a word has been skipped may be a more sensitive measure of mind wandering as it may help differentiate between typical skipping behavior (e.g., skipping short/frequency/predictable words) and atypical skipping behavior due to the dissociation of attention from the text (e.g., overshooting not followed by a corrective regression). Hence, these types of measures may be accurate indicators of the type of mind wandering under investigation in this article, namely instances in which the reader’s attention is decoupled from the text. Indeed, while eye movements during on-task reading have been shown to be impacted by properties of the text (e.g., word-frequency effect), the decoupling of attention from the text may reduce the influence of lexical properties of the text on processing, which may in turn be characterized by a “decoupling” of eye movements and text properties (e.g., a reduced word-frequency effect).

## Conclusion

In this article, we identified two potential indicators of task-unrelated thoughts during reading, namely higher skipping rates and fewer fixations per words. In addition, we identified two types of eye-movement measures that require further investigation as possible indicators of mindless reading, namely readers’ reduced sensitivity to lexical properties of the text, and the use (or lack thereof) of corrective regressions. Lastly, this review identified important methodological questions for future investigations of mindless reading, namely the influence of the chosen window of analysis, as well as how different types of mind wandering are reflected in eye-movement behavior.

## Supplementary Information

Below is the link to the electronic supplementary material.

Supplementary file1 (DOCX 155 KB)

## Data Availability

The data and materials used in the analyses presented in this study are available at https://osf.io/kd8xa/. For the data and materials used in the studies included in the meta-analysis, please consult the articles themselves.

## Footnotes

1 Skipping is a measure that is often reported as “first-pass” skipping, indicating whether a word is skipped during first pass and including words that may be fixated later on during reading. However, this measure was only reported in one article (Reichle et al., 2010) while all other articles calculated skipping rates as words that were never fixated during reading.

## Code availability

The code for the meta-analysis and the additional analyses presented in this article are available at: https://osf.io/kd8xa/
