# Supplementary material for: Eye-movement markers of mind wandering during reading: A meta-analysis
Source: Mem Cognit. 2025 Oct 17;54(3):1064–89. doi: 10.3758/s13421-025-01797-8 (PMC13132886; doi:10.3758/s13421-025-01797-8)
Supplement: Supplementary file 1 — Supplementary file1 (DOCX 155 KB) [file 13421_2025_1797_MOESM1_ESM.docx]

# Eye-Movement Markers of Mind-Wandering during Reading: A Meta-Analysis

**Supplementary Materials
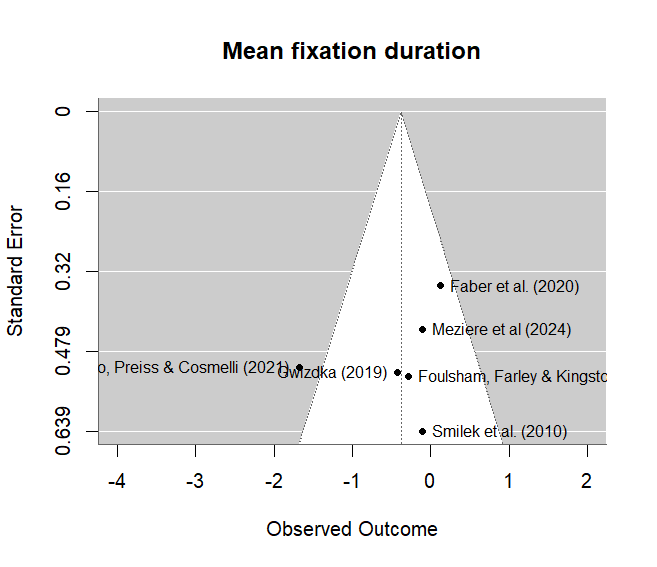
**

**
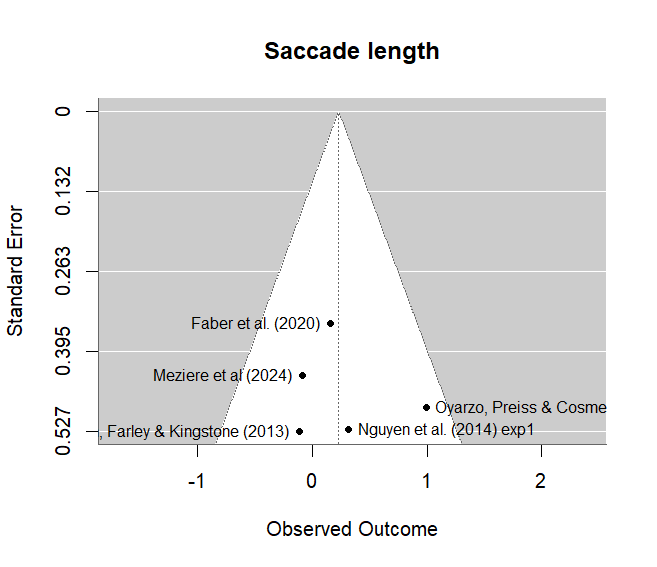

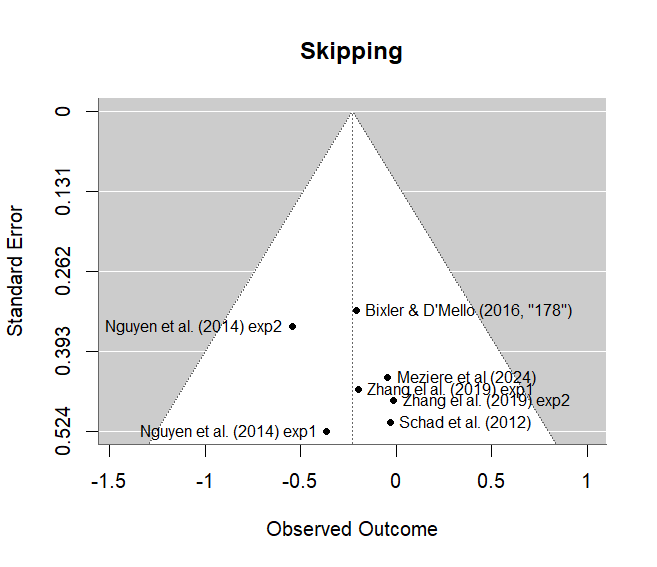
**

**
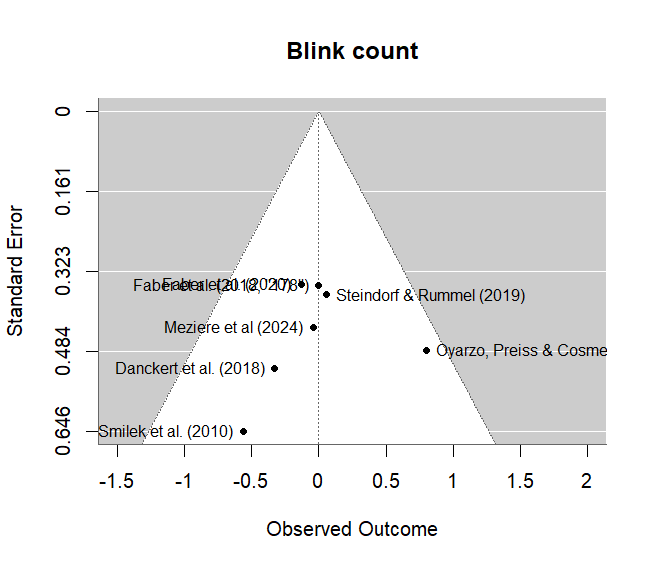

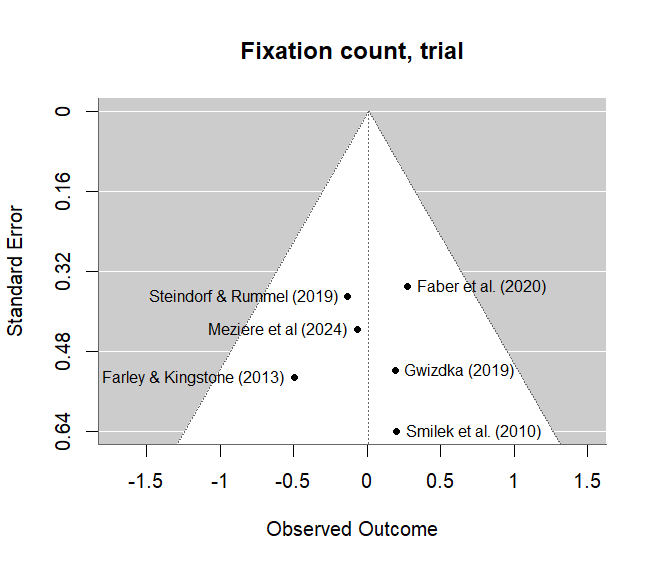
**

**
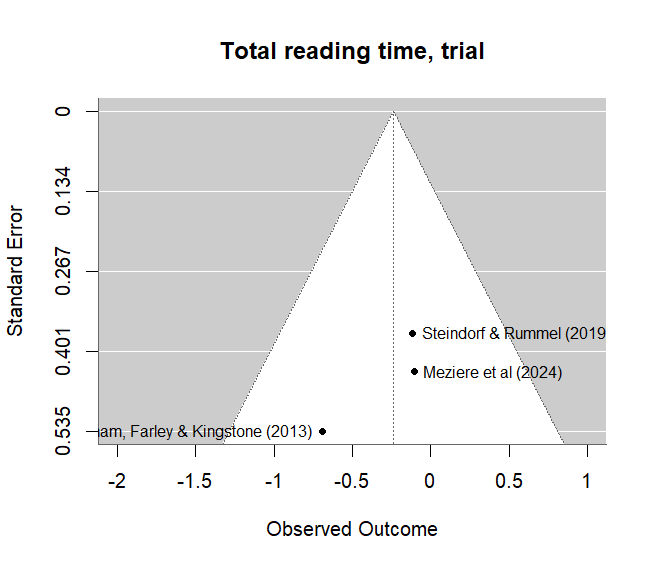
**

**
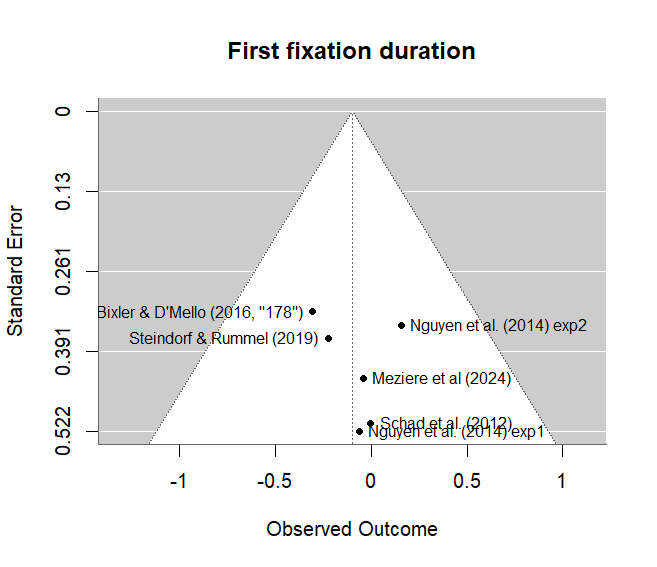
**

**
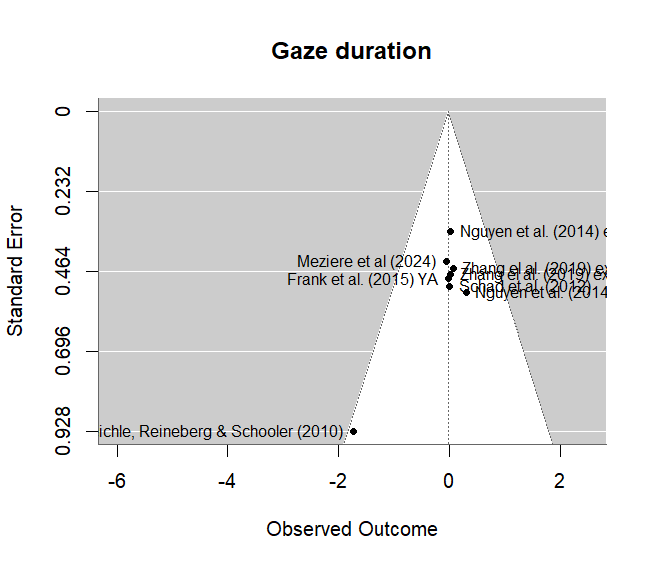
**

**
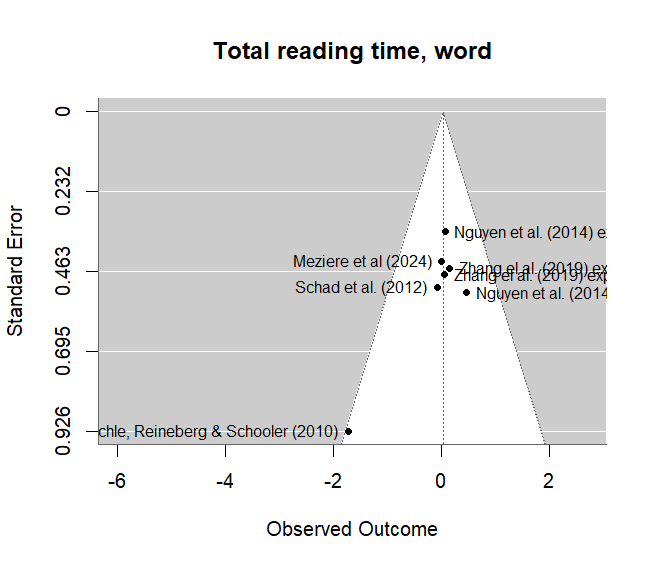
**

**
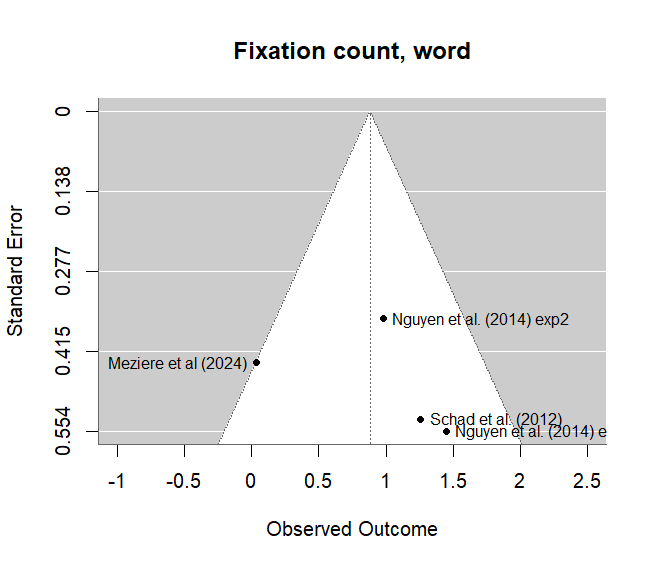
**

**
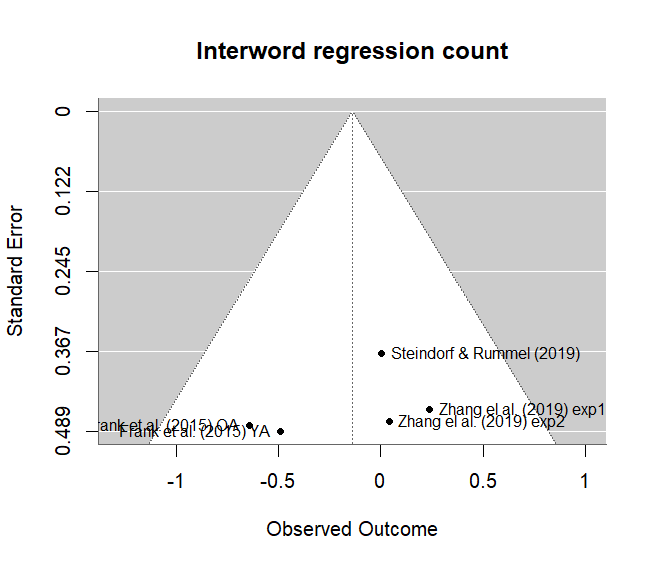
**
